# LONG-TERM NMN TREATMENT INCREASES LIFESPAN AND HEALTHSPAN IN MICE IN A SEX DEPENDENT MANNER

**DOI:** 10.1093/geroni/igad104.3459

**Published:** 2023-12-21

**Authors:** Alice Kane, Karthikeya Chellappa, Michael Schultz, Christian Diener, Sean Gibbons, Joseph Baur, Luis Rajman, David Sinclair

**Affiliations:** Institute for Systems Biology, Seattle, Washington, United States; Brown University, Providence, Rhode Island, United States; Harvard Medical School, Boston, Massachusetts, United States; Institute for Systems Biology, Seattle, Washington, United States; Institute for Systems Biology, Seattle, Washington, United States; Perelman School of Medicine at the University of Pennsylvania, Philadelphia, Pennsylvania, United States; Harvard Medical School, Boston, Massachusetts, United States; Harvard Medical School, Boston, Massachusetts, United States

## Abstract

Levels of nicotinamide adenine dinucleotide (NAD+) decline by up to 50% in aging. NAD+ is an essential co-factor for many metabolic processes, including the deacetylase activity of sirtuins, and previous studies have demonstrated that supplementing NAD+ levels has a range of health benefits in both mice and humans. Here we investigate the effect of long-term (from 13 months of age) administration of the NAD+ precursor nicotinamide mononucleotide (NMN) on frailty and lifespan in male and female mice. NMN treatment delayed the onset of frailty in both sexes, improved metabolic health in male mice, and increased median lifespan by 8.5% in female mice. Exploration of the potential mechanisms of this protection showed that NMN treatment prevented age-related gene expression changes in skeletal muscle and led to a large increase in levels of Anaerotruncus colihominis, a microbe associated with reduced inflammation, in the gut. A thorough characterization of NMN metabolism across age, sex and tissues shows context-specific sex differences in metabolic pathways, including greater Preiss-Handler pathway metabolism in females than males, which may contribute to observed sex differences in health and lifespan. Overall, this data provides preclinical evidence that chronic NMN treatment increases lifespan and improves frailty and metabolic health in aging, and highlights the importance of using both sexes for interventional lifespan studies.

